# Achromobacter xylosoxidans Infection Following Arthroscopic Bankart Repair in an Immunocompetent Patient: A Case Report

**DOI:** 10.7759/cureus.91935

**Published:** 2025-09-09

**Authors:** Soumyadip Sen, Abheek Kar, Abhishek Das

**Affiliations:** 1 Orthopaedics, Apollo Multispeciality Hospitals, Kolkata, IND

**Keywords:** achromobacter xylosoxidans, achromobacter xylosoxidans infection, arthroscopic bankart repair, delayed infection, immunocompetent host infection, post-arthroscopy infection, shoulder infection

## Abstract

Arthroscopic Bankart repair is the most commonly performed procedure for treating anterior shoulder instability. *Achromobacter xylosoxidans*, an aerobic, Gram-negative bacillus, is a rare cause of infection in immunocompetent individuals. Here, we present the case of a 28-year-old male who presented with right shoulder discomfort and a discharging sinus tract eight years after undergoing an arthroscopic Bankart repair. He was treated with arthroscopic debridement and sinus tract excision at another hospital before presenting to us, but the symptoms had recurred within two weeks. Imaging confirmed the presence of a sinus tract communicating with the antero-inferior glenoid rim, with a cystic lesion at the site of the implanted anchors. Under general anesthesia, an open debridement of the right shoulder was undertaken. Osteolysis was noted, and two loose all-suture anchors were removed. Surrounding bone and soft tissue samples were sent for microbiological testing, and antibiotic-impregnated cement beads were placed in the glenoid defect after a thorough lavage. Culture reports revealed *A. xylosoxidans *growth, and culture-directed antibiotics were administered after consulting the infectious disease team. At the two-month follow-up, he was symptom-free, and the sinus tract had completely healed. He had full shoulder function with no signs of infection at the one-year follow-up. This case highlights the need to consider rare, multidrug-resistant pathogens as a cause of postoperative infections, even after minimally invasive procedures in immunocompetent individuals. A high index of suspicion is essential, especially for indolent infections. Early diagnosis, complete hardware removal, and targeted antibiotic therapy are critical for successful outcomes. Additionally, infection with atypical organisms in healthy individuals points toward a probable shift in the pathogenicity of these organisms. To the best of our knowledge, this is the first reported case of *A. xylosoxidans* infection involving the shoulder joint and the first instance of this infection occurring after an arthroscopic procedure.

## Introduction

Arthroscopic Bankart repair has become the gold standard for treating patients with anterior shoulder instability [1,2]. Although it is a safe and secure procedure, recurrent instability, frozen shoulder, and persistence of pain are known complications [2]. Postoperative wound complications and infection are rare, but have been reported in the literature, with an incidence ranging from 0.0% to 0.22% [2-4]. Previously, *Pseudomonas aeruginosa* has been cited as a cause of infection following arthroscopic Bankart repair [5,6].

*Achromobacter xylosoxidans* is an aerobic, non-fermenting, motile, Gram-negative bacillus, which was first isolated in 1971 from chronic otitis media patients [7]. It is a known cause of nosocomial infections in immunocompromised patients and is likely transmitted through intravenous or dialysis fluids [8]. It rarely causes infection in immunocompetent individuals, and musculoskeletal infections have been sparsely reported in the literature [9,10].

To the very best of our knowledge, *A. xylosoxidans* has not been known to cause shoulder joint infection or infections following arthroscopic procedures. Here, we report the case of an indolent *A. xylosoxidans* shoulder infection in a 28-year-old immunocompetent patient, which presented eight years after an arthroscopic Bankart repair surgery.

## Case presentation

A 28-year-old healthy male without any known comorbidities presented to our outpatient department with complaints of discomfort in the right shoulder, associated with a posteriorly discharging sinus tract. He did not have any fever, anorexia, night sweats, or weight loss, and was not on any immunosuppressant medications. Tubercular contact history was negative. He had undergone arthroscopic Bankart repair for recurrent right anterior shoulder instability in 2016 at a different hospital. The symptoms of instability had resolved, and he had achieved a good functional outcome. However, he had mild intermittent discomfort in the shoulder, which was self-resolving.

**Figure 1:**
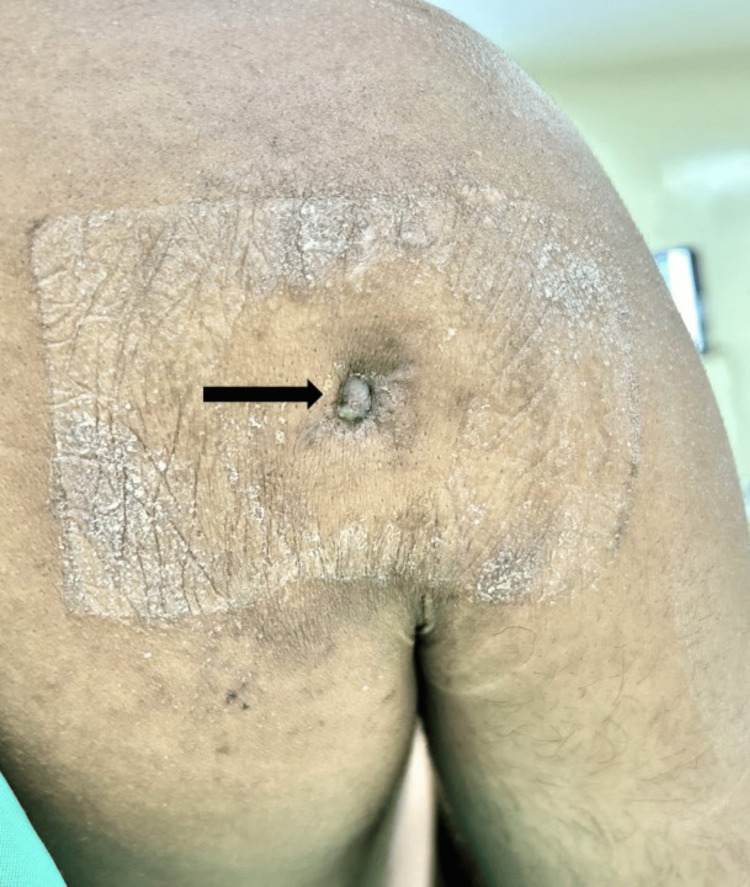
Sinus tract over the posterior aspect of the right shoulder

In June 2024, he had noticed a sensation of wetness on the back of his right shoulder. A discharging sinus tract was noted, and he had undergone an arthroscopic debridement and sinus tract excision at another hospital. The antibiotics administered were not known, as the treatment records were not available. He was symptom-free for two weeks after the procedure, after which he had a recurrence of his symptoms and presented to us. Local examination showed healed scars from the previous surgeries and a sinus tract with scant sero-purulent discharge at the site of the posterior arthroscopic viewing portal (Figure 1). The local temperature was raised, but there was no swelling or erythema. Shoulder movements were painless and comparable to those on the opposite side; the apprehension test was negative, and he had no history of other hospitalizations. The American Shoulder and Elbow Surgeons (ASES) score for the right shoulder was 87 [11].

**Figure 2:**
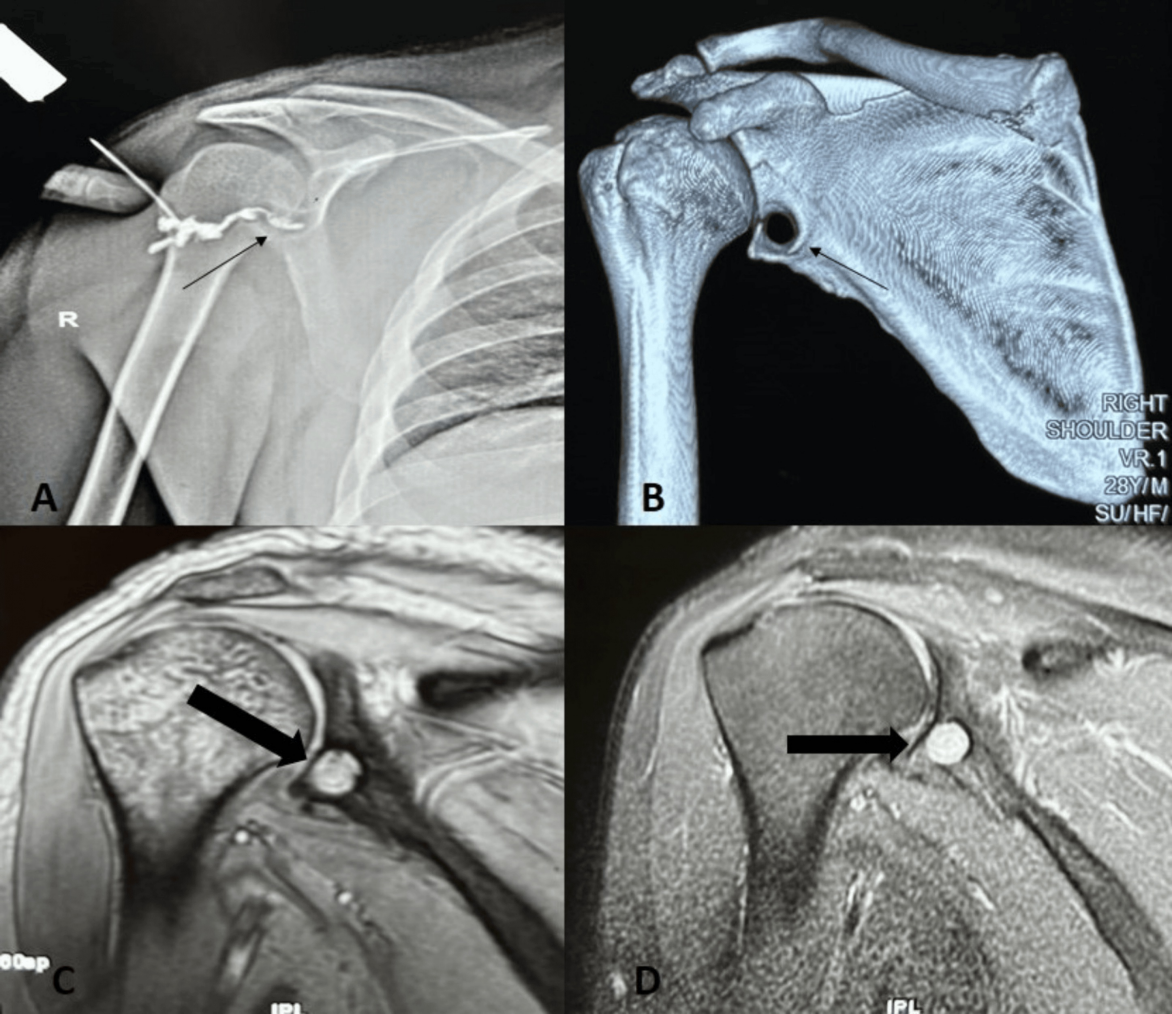
Pre-operative images A: Sinogram showing the sinus tract communicating with the antero-inferior glenoid rim. B: Computed tomography scan showing a well-corticated defect in the antero-inferior glenoid. C: T1-weighted magnetic resonance image showing a hypointense lesion. D: T2-weighted magnetic resonance image showing a hyperintense lesion.

Laboratory investigations were non-revealing except for a raised erythrocyte sedimentation rate (ESR), which was 84 mm in the first hour. The total leukocyte count was 8.3x10^9^/L, and the C-reactive protein (CRP) level was 0.3 mg/dL. A sinogram revealed the sinus tract to be communicating with the antero-inferior glenoid rim (Figure 2A). A non-contrast computed tomography (CT) scan showed a well-corticated linear defect in the antero-inferior aspect of the glenoid (Figure 2B). On magnetic resonance imaging (MRI), a well-defined, rounded cystic lesion was noted, which appeared hypointense on T1-weighted images and hyperintense on T2-weighted images, consistent with a fluid-filled cavity, without any significant gleno-humeral joint effusion (Figures 2C, 2D). A swab culture from the sinus discharge showed no growth of organisms, and he refused to undergo a shoulder joint aspiration. The in-situ suture anchors were the likely source of persistent infection. Treatment options were discussed with the patient, and an open debridement of the right shoulder with probable removal of the suture anchors was planned.

**Figure 3:**
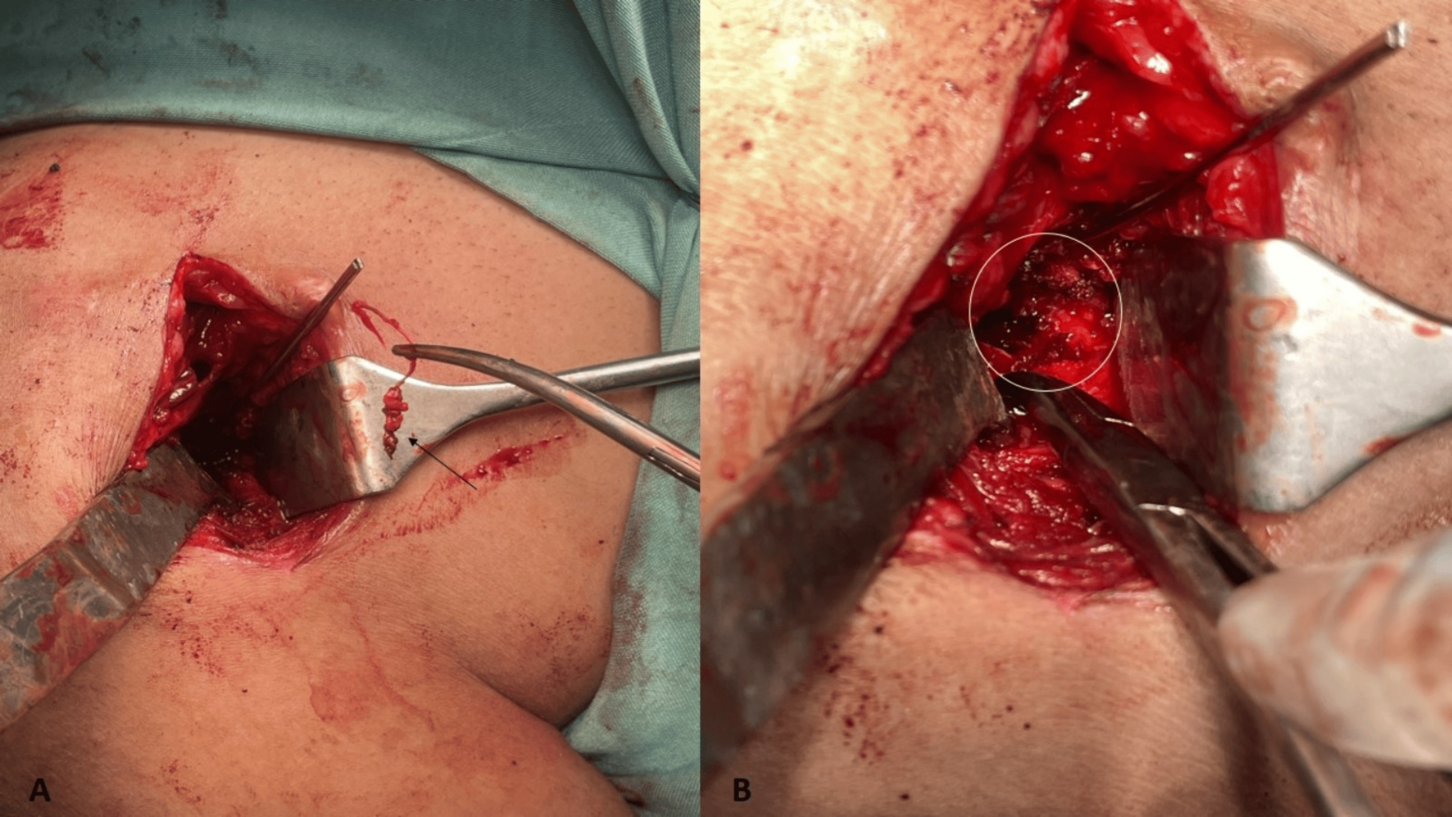
Intraoperative images A: Loose anchor that was removed. B: Cavity in the antero-inferior aspect of the glenoid.

Under general anesthesia, the right shoulder joint was exposed by the deltopectoral approach in the beach chair position. A sterile dressing was used to cover the sinus tract during the procedure. After exposing the gleno-humeral joint, a Fukuda retractor was used to retract the humeral head. Some osteolysis was noted at the glenoid rim, and two loose all-suture anchors were removed (Figure 3A). Surrounding bone and soft tissue samples were sent for microbial culture and sensitivity testing. The anchor site was thoroughly scraped out (Figure 3B). No additional shoulder stabilization procedure was done as the joint was stable. After a thorough wound lavage, absorbable synthetic calcium sulfate cement beads impregnated with 40 mg of gentamicin and 1 g of vancomycin were placed in the glenoid defect left by the anchors. The wound was sutured in layers. The sinus tract was not excised due to the proximity of surrounding neurovascular structures and the need for extensive tissue dissection. As the inciting foreign material had been removed, it was expected to heal by itself.

**Figure 4:**
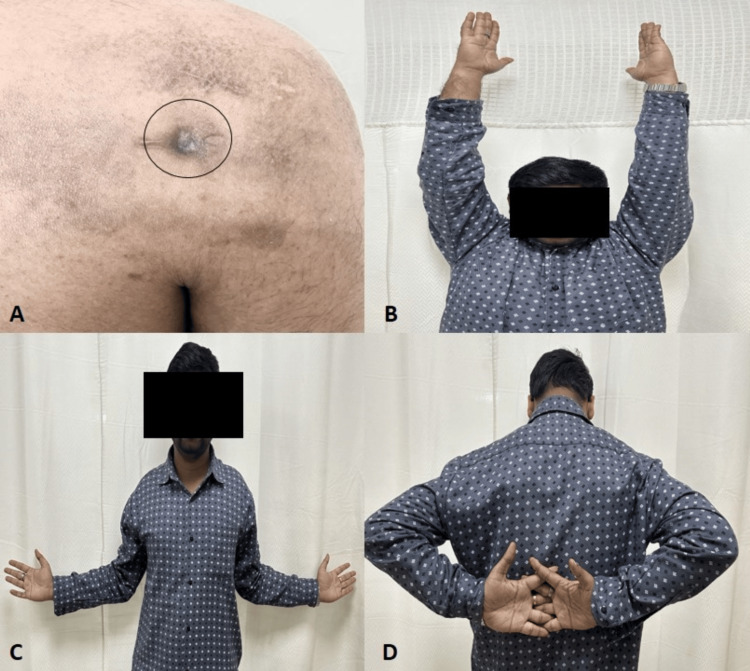
Follow-up clinical examination A: Healed sinus tract. B: Forward flexion. C: External rotation. D: Internal rotation.

Shoulder mobilization, as tolerated by our patient, was allowed postoperatively. Intravenous cefuroxime was given twice daily. After four days, microbial culture revealed *A. xylosoxidans* growth, and intravenous cefoperazone-sulbactam was administered instead of cefuroxime for two weeks. After consultation with the infectious disease team, he was then switched to 65 mg extended-release oral minocycline daily for a further six weeks. At the two-month follow-up, he was symptom-free. The sinus tract had healed, and no collection could be expressed (Figure 4A). The ESR values had returned to normal (8 mm in the first hour). At one-year follow-up, he had full shoulder function with no residual pain or signs of infection (Figures 4B-4D). The ASES score had improved to 100 [11].

## Discussion

Arthroscopic Bankart repair has surpassed open repair as the preferred treatment for shoulder instability due to the distinct advantages offered by it, such as less invasiveness, enhanced diagnostic ability, lower risk of stiffness, and avoidance of biceps tenotomy and subscapularis splitting [2-4]. Intraoperative and postoperative complication rates are minimal [2]. Although there have been reports of infection following arthroscopic Bankart repair, infections involving the deep tissue or the glenohumeral joint are very rare [1,2].

The organisms that are known to cause infections after shoulder arthroscopic procedures include *Staphylococcus epidermidis*, *Staphylococcus aureus,* *Cutibacterium acnes*, and *P. aeruginosa* [6,12]. Rare cases due to *Mycobacterium tuberculosis* and *Moraxella catarrhalis* have also been reported [13]. To the best of our knowledge, no cases of shoulder joint infection due to *A. xylosoxidans* have been previously documented in the available literature.

*A. xylosoxidans* is an opportunistic pathogen predominantly causing nosocomial infections in immunocompromised individuals [7,8,14,15]. It is found in aqueous environments and is thought to be transmitted via intravenous or dialysis fluids in nosocomial infections [7,8,15]. Otitis media, meningitis, urinary tract infection, endocarditis, peritonitis, pneumonia, endophthalmitis, keratitis, and bacteremia are some of the reported infections caused by it [7-10,14]. Osteomyelitis, septic arthritis, and prosthetic joint infections are some of the known osteoarticular infections [7,8,14,16,17]. Though the majority of infections occurred in immunocompromised patients, two cases each of prosthetic joint infection and osteomyelitis, and one case of septic arthritis were reported in immunocompetent individuals (Table 1) [9,10,14,15,18].

**Table 1:** Summary of osteoarticular infections due to Achromobacter xylosoxidans in immunocompetent patients

Primary author (year)	Age/gender	Site of infection	Preceding surgery or trauma	Time to presentation	Management	Outcome
Present case	28 years/male	Right shoulder	Arthroscopic Bankart repair	8 years	Open debridement, anchor removal, joint lavage	Symptom-free at 1 year
Ojha et al. (2024) [14]	61 years/female	Prosthetic joint infection of right knee	Bilateral total knee arthroplasty	1 year	Two-stage revision arthroplasty	Symptom-free at 3 months
Imani et al. (2021) [18]	23 years/male	Chronic osteomyelitis of right femur	Right femur intramedullary fixation	5 years	Debridement with reamer-irrigator-aspirator	Symptom-free at 1 year
Suryavanshi et al. (2015) [10]	1 month and 11 days/male	Septic arthritis of left hip	None	Not relevant	Emergency hip arthrotomy	No deformity or symptoms on subsequent visits
Pamuk et al. (2015) [15]	15 years/female	Osteomyelitis of right talus, navicular, and cuneiform; septic thrombophlebitis of great saphenous vein	Purulent discharge from right ankle lesion	1 week	Meropenem, amikacin, and vancomycin for four weeks	Complete recovery
Lee et al. (2014) [9]	52 years/male	Prosthetic joint infection of right knee	Right total knee arthroplasty	First presentation 4 weeks after surgery; sinus tract formation 18 months after surgery	First procedure: sinus tract debridement; second procedure: two-stage revision arthroplasty	Symptom-free at 12 months

In suspected cases of postoperative infection, synovial fluid culture is commonly performed to detect the causative organism, although it may be inadequate to detect the pathogen in some cases [1]. It may be prudent to administer empirical intravenous or oral antibiotics as soon as an infection is suspected, which can be subsequently tailored as per culture and sensitivity reports [1]. If antibiotics alone are insufficient for infection control, operative treatment such as synovectomy, joint lavage, and drainage can be performed [1,5]. Additional removal of sutures and/or anchors can be done for peri-anchor infections [1]. Postoperative administration of culture-directed oral or intravenous antibiotics for four to six weeks is recommended for complete eradication [5,6].

Although arthroscopic Bankart repair has a minimal infection rate, cases with *P. aeruginosa* infection necessitating debridement and anchor removal have been reported (Table 2) [5,6]. Ticker et al. reported the first case of infection surrounding implanted suture anchors in 1996, which occurred due to *S. aureus* following an open Bankart repair [19]. In all these cases, signs of infection appeared within the first two months following surgery, whereas our patient presented eight years after the index procedure. The previously reported patients had been treated with metallic and polyetheretherketone (PEEK) anchors, while all-suture anchors were retrieved from our patient [5,6,19].

**Table 2:** Summary of shoulder infections following arthroscopic Bankart repair PEEK: polyetheretherketone

Primary author (year)	Age in years/gender	Clinical features	Time to presentation	Causative pathogen	Type of anchor	Management	Outcome
Present case	28/male	Right shoulder discomfort with discharging sinus tract	8 years	*Achromobacter* xylosoxidans	All-suture anchor	Open debridement, suture anchor removal, joint lavage	Symptom-free at 1 year
Mohan et al. (2022) [5]	54/male	Red, non-tender swelling of left shoulder with a sinus	4 weeks	Pseudomonas aeruginosa	PEEK anchor	Arthroscopic debridement, lavage, and anchor removal	Symptom-free at 1 year
Kumar et al. (2016) [6]	22/male	Fever, left shoulder pain, and swelling	2 months	P. aeruginosa	Metal anchor	Arthroscopic debridement and anchor removal	Symptom-free at 1 year

*A. xylosoxidans* is resistant to multiple antibiotics, including aminoglycosides (due to aminoglycoside-modifying enzymes), first- and second-generation cephalosporins (due to plasmid-mediated beta-lactamases), and some fluoroquinolones [7,8,18]. Hence, treatment with empiric antibiotic therapy can be quite challenging without prior knowledge of the causative organism and its antimicrobial susceptibility. Incomplete eradication of bacterial reservoirs can be a source for recurrent infections [8]. Our patient was treated with culture-directed antibiotics. Minocycline was chosen as it had a lower minimum inhibitory concentration, and it could also be administered orally with a once-daily dosing, which ensured better patient compliance.

We speculate that the disease might have been contracted due to contaminated irrigation fluids during the index procedure, and it had run an indolent course over the past eight years, as our patient was immunocompetent. The sinus tract may have developed posteriorly due to the gravity-dependent fluid accumulation and subsequent drainage along the posterior portal site. Other signs of infection, such as fever, swelling, or erythema, were absent. An arthroscopic irrigation and debridement of the shoulder joint is considered more appropriate compared to an open procedure, as it has been shown to have lower lengths of hospital stay, reduced blood transfusions, reduced wound complications, and lower rates of revision irrigation and debridement surgeries [5,20]. However, an arthroscopic debridement procedure performed previously at another center had failed to completely eradicate the infection in our patient; hence, a thorough open debridement with anchor removal was performed.

## Conclusions

A high degree of suspicion is needed to identify infection in patients presenting with subclinical symptoms after arthroscopic Bankart repair. *A. xylosoxidans* is prevalent in aquatic habitats and is a known cause of nosocomial infections. It can be a possible cause of infection even after minimally invasive procedures in immunocompetent individuals.

Removal of the harbor of infection, such as any implanted hardware, adequate debridement, and culture-directed antibiotic therapy, should be the preferred treatment. This also highlights a potential shift in the infectivity and behavior of environmental organisms, reflecting an evolving trend in microbial pathogenicity, even among typically low-virulence species.

## References

[1] Matsuki K, Sugaya H (2015). Complications after arthroscopic labral repair for shoulder instability. Curr Rev Musculoskelet Med.

[2] Rodriguez K, Hurley ET, Park CN (2024). Complications following arthroscopic Bankart repair: a systematic review. J Shoulder Elbow Surg.

[3] Owens BD, Harrast JJ, Hurwitz SR, Thompson TL, Wolf JM (2011). Surgical trends in Bankart repair: an analysis of data from the American Board of Orthopaedic Surgery certification examination. Am J Sports Med.

[4] Eberlin CT, Varady NH, Kucharik MP, Naessig SA, Best MJ, Martin SD (2022). Comparison of perioperative complications following surgical treatment of shoulder instability. JSES Int.

[5] Mohan PK, Nair AV, Kuntwad V, Jangale A, Kumar MP, Adhikary R (2022). Indolent and delayed presentation of Pseudomonas aeruginosa infection of the shoulder joint after arthroscopic Bankart repair and remplissage surgery: a case report. Int J Orthop Sci.

[6] Kumar M, Thilak J (2016). Infected shoulder joint with loose suture anchor in the joint after Bankart's repair- a case report. J Orthop Case Rep.

[7] Shinha T, Oguagha IC (2015). Osteomyelitis caused by Achromobacter xylosoxidans. IDCases.

[8] Patel PK, von Keudell A, Moroder P, Appleton P, Wigmore R, Rodriguez EK (2015). Recurrent septic arthritis due to Achromobacter xylosoxidans in a patient with granulomatosis with polyangiitis. Open Forum Infect Dis.

[9] Lee SC, Nam CH, Park IS, Yoon JY, Jung KA, Hwang SH (2014). Achromobacter xylosoxidans infection following total knee arthroplasty. J Korean Orthop Assoc.

[10] Suryavanshi KT, Lalwani SK (2015). Uncommon pathogen: serious manifestation: a rare case of Achromobacter xylosoxidans septic arthritis in immunocompetetant patient. Indian J Pathol Microbiol.

[11] Tan JH, Teo E, Kumar VP, Poh KS, Lim JL (2025). 1-year postoperative follow-up is adequate for arthroscopic Bankart repair: a comparison between 1-year and 2-year postoperative patient-reported outcome scores. JSES Int.

[12] Pauzenberger L, Grieb A, Hexel M, Laky B, Anderl W, Heuberer P (2017). Infections following arthroscopic rotator cuff repair: incidence, risk factors, and prophylaxis. Knee Surg Sports Traumatol Arthrosc.

[13] Kim YB, Kim J, Song MG, Kim TH, Choi TY, Seo GW (2024). Glenohumeral joint septic arthritis and osteomyelitis caused by Moraxella catarrhalis after arthroscopic rotator cuff repair: case report and literature review. J Bone Jt Infect.

[14] Ojha MM, Ansari MA, Janardhanan R, Kumar V (2024). Uncommon pathogen: Achromobacter xylosoxidans infection following total knee arthroplasty. J Orthop Case Rep.

[15] Pamuk G, Aygun D, Barut K, Kasapcopur O (2015). Achromobacter causing a thrombophlebitis and osteomyelitis combination: a rare cause. BMJ Case Rep.

[16] Girgis CP, Coye TL, Ansert E, Killeen AL, Crisologo PA (2025). Rare case of osteomyelitis caused by Achromobacter xylosoxidans. J Am Podiatr Med Assoc.

[17] Singh A, Hussain A, Jain R, Aishwarya K, Tak V, Thakur P (2017). Achromobacter xylosoxidans septic arthritis in a child with primary immunodeficiency. J Glob Infect Dis.

[18] Imani S, Wijetunga A, Shumborski S, O'Leary E (2021). Chronic osteomyelitis caused by Achromobacter xylosoxidans following orthopaedic trauma: a case report and review of the literature. IDCases.

[19] Ticker JB, Lippe RJ, Barkin DE, Carroll MP (1996). Infected suture anchors in the shoulder. Arthroscopy.

[20] Upfill-Brown A, Shi B, Carter M (2022). Lower risk of revision surgery after arthroscopic versus open irrigation and Débridement for shoulder septic arthritis. J Am Acad Orthop Surg.

